# Effects of dual-task training in patients with post-stroke cognitive impairment: A randomized controlled trial

**DOI:** 10.3389/fneur.2022.1027104

**Published:** 2022-10-24

**Authors:** Ruifeng Sun, Xiaoling Li, Ziman Zhu, Tiancong Li, Man Zhao, Linhong Mo, Wenshan Li, Xiaoshuang Xi, Peiling Huang, Weijun Gong

**Affiliations:** ^1^Beijing Rehabilitation Medicine Academy, Capital Medical University, Beijing, China; ^2^Department of Neurological Rehabilitation, Beijing Rehabilitation Hospital, Capital Medical University, Beijing, China

**Keywords:** post-stroke cognitive impairment, cognitive-motor dual task, randomized controlled trial, event-related potential, functional near-infrared spectroscopy

## Abstract

**Background:**

Evidence for the efficacy of cognitive-motor dual-task (CMDT) training in patients with post-stroke cognitive impairment (PSCI) and no dementia is still lacking. More importantly, although some studies on the cognitive effect of CMDT training show an improvement in cognitive performance, the results are still controversial, and the intervention mechanism of CMDT training on cognitive function improvement is not clear. The main purpose of this study was to analyze the effects of CMDT training on cognitive function, neuron electrophysiology, and frontal lobe hemodynamics in patients with PSCI.

**Methods:**

Here we tested the effects of CMDT training on cognitive function in PSCI patients. Forty subjects who met the criteria of PSCI were randomly assigned to control and experimental groups. CMDT training or cognitive task (CT) training was administered to each patient in the experimental and control groups, respectively. All subjects performed Mini-mental State Examination (MMSE) and Montreal Cognitive Assessment (MoCA) scale before and after the intervention, and the event-related potentials (ERP) and functional near-infrared spectroscopy (fNIRS) were used to evaluate the changes in neuron electrophysiology and hemodynamics.

**Results:**

Forty patients were randomized across Beijing Rehabilitation Hospital Capital Medical University in Beijing. At the end of the intervention, 33 subjects completed the experimental process. The CMDT group showed significant improvement in the MMSE (*P* = 0.01) and MoCA (*P* = 0.024) relative to the CT group. The results of ERP and fNIRS showed that CMDT training could shorten the latency of P300 (*P* = 0.001) and the peak time of oxygenated hemoglobin (*P* = 0.004). The results showed that CMDT training shortened the response time of central neurons and significantly increased the rate of oxygen supply to the frontal lobe.

**Conclusion:**

CMDT training in patients with PSCI improved global cognitive function, which was supported by the improved neural efficiency of associated brain areas.

**Clinical trial registration:**

http://www.chictr.org.cn, identifier ChiCTR2000034862.

## Introduction

Stroke is one of the leading causes of death and adult disability, and it is highly associated with an increased risk of cognitive impairment (1). The Stroke and Cognition Consortium (STROKOG) harmonized data from 13 studies based in eight countries found that 44% of hospitalized stroke participants were impaired in global cognition (2). In China, a community-based study involving 599 stroke patients showed that the incidence of post-stroke cognitive impairment (PSCI) was as high as 81.0%, with 48.9% of cases being PSCI patients with non-dementia and 32.1% being post-stroke dementia patients (3). PSCI is a broad concept that covers the full spectrum from mild cognitive impairment to dementia and includes cases with solely vascular pathology or mixed pathologies (4). It refers to a series of syndromes that meet the diagnostic criteria of cognitive impairment after stroke, emphasizing the potential causal relationship between stroke and cognitive impairment and the correlation of clinical management between them (5, 6). The active clinical management of stroke may reduce the occurrence of PSCI, and the intervention of PSCI may also contribute to better recovery from stroke dysfunction.

Previous studies have shown that cognitive training (CT), including cognitive behavioral training and computerized cognitive training, can improve cognitive impairment by enhancing the functional connection of related brain regions and cerebral cortical activity and increasing the thickness and volume of the cerebral cortex (7, 8). Exercise training has a beneficial effect on cognitive function by increasing cerebral blood flow and reducing cardio-cerebrovascular risk factors (9, 10). But traditional cognitive rehabilitation training has many limitations in terms of efficacy and implementation. At present, it is suggested that cognitive-motor dual-task (CMDT) training may have a synergistic effect on improving cognitive function (11). A network meta-analysis by Gavelin et al. (12) shows that combined intervention can effectively improve the cognitive function of the elderly with and without cognitive impairment. The impact of CMDT training on cognitive function has been studied extensively and some creative points need to be addressed. Due to the lack of high-quality randomized controlled trials (RCT) studies, the outcome of combined intervention in the improvement of cognitive function in PSCI patients is not consistent. More importantly, the neurophysiological manifestations and cerebral blood flow changes of CMDT training are not clear.

Event-related potential (ERP) is a neuron electrophysiology detection method for the cognitive function of the brain (13). The generation and changes of EEG can express the activity process of brain cells in real-time, and the ERP can record the cognitive processing in real-time (14). It has the advantages of high time resolution, real-time, objectivity, popularity, and so on. ERP including P300, N200, MMN, and other different components can be used in the detection of cognitive impairment, among which P300 is the most classic and widely used. P300 is an endogenous component of ERP, which is not affected by external stimuli and can objectively evaluate the electrical activity process of the brain (15). Studies have shown that the main manifestation of P300 in patients with vascular cognitive impairment is prolonged latency and no significant decrease in amplitude, indicating that prolonged latency is a more characteristic manifestation (16).

The current research provides strong evidence that neurovascular coupling plays a causal role in the pathogenesis of vascular cognitive impairment. Neurovascular coupling is a critical homeostatic mechanism in the brain, responsible for the adjustment of local cerebral blood flow to the energetic needs of the active neuronal tissue (17). Functional near infrared spectroscopy (fNIRS) is a new non-invasive brain function monitoring technique. Its spatial resolution is better than that of EEG, and its time resolution is better than that of magnetic resonance technology. JÖBSIS first used f-NIRS to observe the changes in blood oxygen content in the brain of animals in 1977 (18). The principle of f-NIRS is to detect the light source energy after scattering by using the good permeability of near-infrared light to human tissue. The hemodynamic changes of the cerebral cortex were reflected by calculating the concentration of oxygenated hemoglobin(oxy-Hb), deoxyhemoglobin, and total oxygen (19). This indirectly reflects the activation and functional changes of the relevant brain regions.

Thus, we conducted a proof-of-concept single-blind RCT study to provide evidence of the efficacy of CMDT training for cognitive function improvement among patients with PSCI. At the same time, we used ERP and fNIRS to explore a potential neural mechanism for cognitive functional changes.

## Materials and methods

### Study design, registration, and patient consent

The study included three parts: Baseline measurements, a 4 week intervention phase, and measurements at the end of the intervention period. Participants with PSCI were recruited from Beijing Rehabilitation Hospital at Capital Medical University. Ethical approval was obtained from the Ethics Committee of Beijing Rehabilitation Hospital at Capital Medical University (2020ky59). The trial was registered under chictr.org.cn (ChiCTR2000034862). All the participants provided written informed consent.

### Participants

Adults with a clinical diagnosis of PSCI were included in the study (20). All patients met the following inclusion criteria: (1) patients with the first stroke and lesions located in the cerebral hemisphere; (2) ages 18–80; (3) cognitive function evaluation: informant questionnaire on cognitive decline in the elderly (IQCODE) ≤ 3.3, 9 < Mini-mental State Examination (MMSE) < 27, and 9 < Montreal Cognitive Assessment (MoCA) < 26; (4) able to follow the learning guide clearly; (5) walking function: Holden grade > 2, orthostatic balance ≥ 2; (6) attending physician evaluated the cardiopulmonary function of the subjects and determined that they could complete the aerobic exercise. Exclusion criteria were as follows: (1) participants who exhibited disorders other than PSCI that would affect cognition; (2) taking drugs that would affect cognitive impairment; (3) clinically significant gastrointestinal, renal, hepatic, respiratory, or other systemic diseases; (4) severe deafness, blindness, or major physical diseases that led to communication disorders and made the patients unable to participate in screening; (5) seriously impaired limb function that made the patients unable to complete the corresponding exercise training; (6) participation in other trials or other treatments at the same time.

### Randomization and blinding

We used the online Research Randomizer to generate the allocation sequence and used block randomization to achieve two groups with a 1:1 ratio. The personnel involved in conducting the study and data analysis were masked to the patient randomization. All evaluators and data analysts were blinded to treatment assignment throughout the study.

### Sample size

There were two groups in the study, the CMDT group, and the CT group. We used G^*^Power software 3.1 prior power analysis to estimate the sample size (α = 0.05, and 1 – β = 0.8). No previous randomized trial had examined the effect of CMDT training on cognitive function in PSCI. We chose an effect size of 0.4, and the calculated minimum sample size was 34 cases. Assuming that the drop-off rate of the whole experiment was 10%, the total sample size was calculated to be 38 patients with PSCI. The patients were randomly divided into two groups with a 1:1 ratio, and the final number of patients in each group should be 19. Finally, a total of 33 participants in the CMDT group and CT group were included in our study because seven patients dropped out during the study.

### Procedures

Patients in the CT group received an individualized multidomain progressive training program for 4 weeks. Participants were required to complete 40 min of training per day, 5 days a week. Within each domain, high accuracy (>90%) was required to upgrade to the next difficulty level (7). The training domains are determined according to the results of cognitive evaluation before training, including calculation, reasoning, working memory, processing speed, executive control, and attention. Patients in the CMDT group received simultaneous cognitive training and motor training. The content of cognitive training was the same as that of the CT group. Exercise training included 20 min of rehabilitation treadmill training (plus tolerable resistance) and walking on a flat surface for 20 min (at a speed acceptable to the patient). The patients' attention had to be allocated to both motor tasks and cognitive tasks. The intervention began directly after randomization. All the outcomes were assessed at the baseline and end of intervention after randomization to measure the effect.

### Outcomes

The primary outcome measures were global cognitive function, measured by MMSE and MoCA. Based on previous studies, we hypothesized that CMDT training could enhance neural reactivity and increase cerebral blood flow. The secondary outcomes of the present study, therefore, included the amplitude and latency of P300, a key indicator linked with neural reactivity (21), and the changing trend of oxy-Hb concentration, which is associated with cerebral blood supply.

### ERP data

The ERP data were acquired *via* the classical oddball paradigm. The patients sat in front of the computer screen that presented the cognitive task and placed a healthy hand on the mouse. The screen randomly showed the number 2 or 8. The standard stimulus and deviation stimulus were displayed 70 and 30% of the time randomly. The number two was the target stimulus. Before training, it was explained to the patient that they should press the left mouse button when the screen showed 2 and not press anything when the screen showed 8. The whole test lasted 2 min. The analysis of the P300 components included the presence of latency and amplitude. The P300 components were obtained at the Fz electrode sites. The P300 latency was identified manually in the time window of 300–700 ms and amplitude was defined as the maximum peak within the same time window.

### fNIRS data

**Figure 1:**
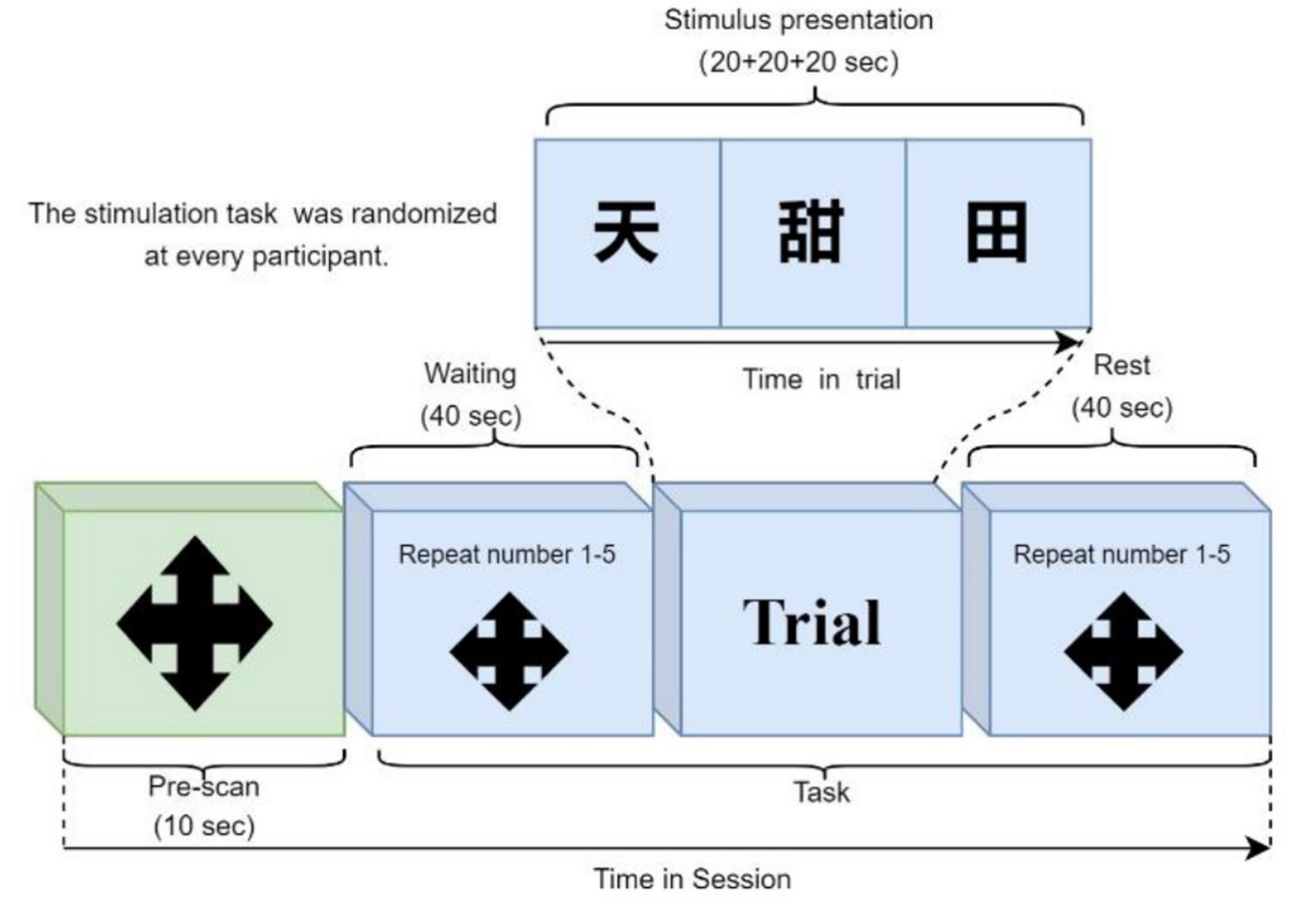
VFT task-based experiment protocol.

The fNIRS data were acquired *via* the ETG-4000 Optical Topography system. The mode was continuous wave technology, and the changing trend of oxy-Hb concentration was measured. The acquisition frequency of the fNIRS signal was 10 Hz, and the measuring depth was 2.0–3.0 cm. During the experiment, all participants fixated on the screen to perform the verbal fluency (VFT) task (Figure 1). Before measuring fNIRS signals, an introduction and exercises for the VFT task were preceded to ensure participants fully understood the task. After the introduction and exercises, each participant performed a VFT task session. The session consisted of three trials. Trials consisted of three different characters with the same pronunciation (20s / a Chinese character). Record the reaction time and operation accuracy of all participants for data analysis.

### Statistical analyses

Statistical analysis was performed using the SPSS 22.0 software package (IBM Corporation, Armonk, NY). The significance of the difference between the two groups of variables was determined by the *t*-test and described by mean (standard deviation), with the measurement data that did not conform to the normal distribution analyzed using the Mann–Whitney U test and described by median (quartile spacing). A statistically significant difference was *P* < 0.05.

## Results

### Participants' characteristics

**Figure 2:**
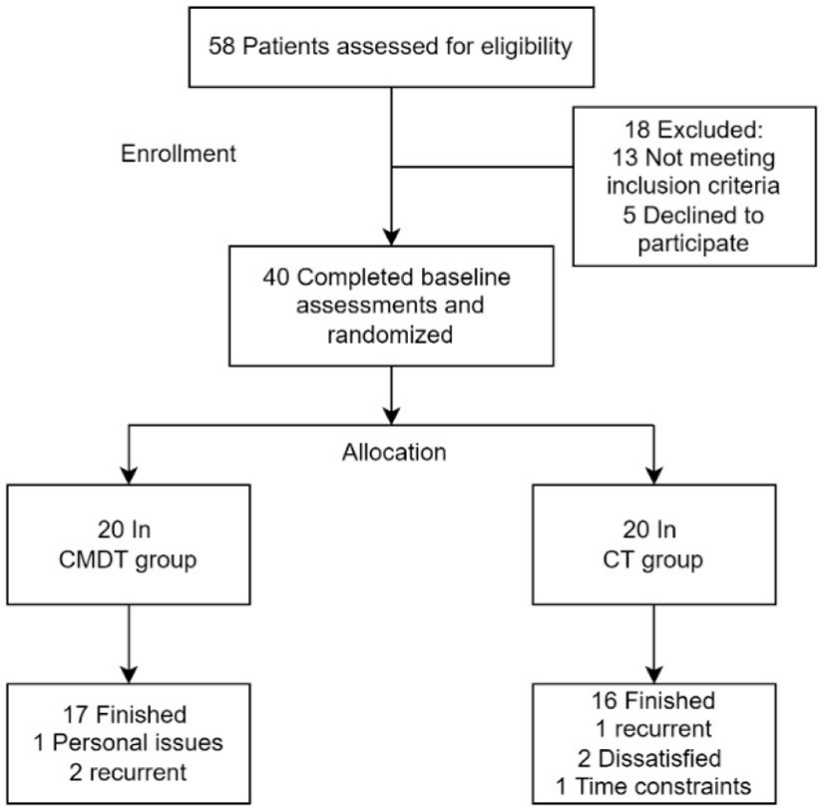
The flowchart for this study.

Forty individuals were recruited between November 2019 and March 2021. The flow of participants through the study is shown in Figure 2. Baseline characteristics and neuropsychological assessment data are shown in Table 1. We found no differences in age, sex, or duration of education. The average course of disease (SD) in the dual task group and cognitive training group was 3.2 (1.1) months and 3.1 (1.089) months respectively, which was in the middle stage of stroke recovery. The neuropsychological testing scores were matched between the two groups. Concerning their neuropsychological test performance, both groups performed comparatively (*P* = 0.301–0.336). All participants completed more than 90% of the training requirement.

**Table 1:** Baseline characteristics.

**Variables**	**CMDT** **(*n* = 17)**	**CT** **(*n* = 16)**	**P-value**
Age (yr), mean (SD)	54.0 (14.0)	60.5 (10.6)	*P =* 0.159
Sex, *n* females (%)	5 (29.4)	3 (18.8)	*P =* 0.688
BMI, mean (SD)	25.7 (2.5)	24.8 (3.9)	*P =* 0.121
Education (yr), mean (SD)	12.9 (3.8)	13.3 (2.7)	*P =* 0.723
Type, *n*% (I/H)^*^	11 (64.7)/6 (35.3)	12 (75)/4 (25)	*P =* 0.708
Course (month), mean (SD)	3.2 (1.1)	3.1 (1.089)	*P =* 0.940
ADL, mean (SD)	75.0 (6.58)	75.9 (7.55)	*P =* 0.794
MMSE _baseline_	21.8 (3.0)	22.9 (2.6)	*P =* 0.336
MoCA _baseline_	17.9 (3.4)	19.4 (3.2)	*P =* 0.301

SD, Standard Deviation; BMI, Body Mass Index; Course, course of the disease; ADL, Activity of Daily Living Scale; MMSE, Mini-Mental State Examination; MoCA, Montreal Cognitive Assessment;

* I, infarction; H, hemorrhage.

### Primary index measurement

**Figure 3:**
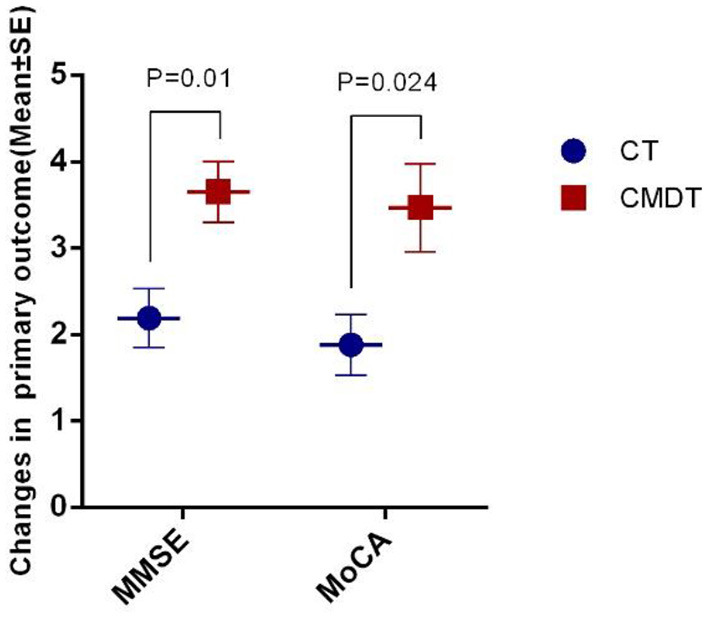
Training effect on the primary outcome measurement.

Table 2 shows the covariate-adjusted change from baseline to 4 weeks (end of intervention) for the two primary outcome variables. After 4 weeks, the patients in the CMDT group had significantly improved compared to the CT group, as measured by MMSE (*P* = 0.01) and MoCA (*P* = 0.024) (Figure 3).

**Table 2:** Estimated mean change (SD) and statistical significance in primary outcome variables.

**Variables**	**Group**	**Baseline**	**Outcome**	**Difference**	**Z**	***P*** **-value**
MMSE	CMDT	21.76 (2.95)	25.41 (2.92)	3.65 (1.46)	−2.587	*P =* 0.01^*^
	CT	22.88 (2.63)	25.06 (2.64)	2.19 (1.38)		
MoCA	CMDT	17.94 (3.41)	21.41 (3.83)	3.47 (2.10)	−2.251	*P =* 0.024^*^
	CT	19.44 (3.20)	21.31 (3.22)	1.88 (1.41)		

* Statistical significance.

### Secondary index measurement

**Figure 4:**
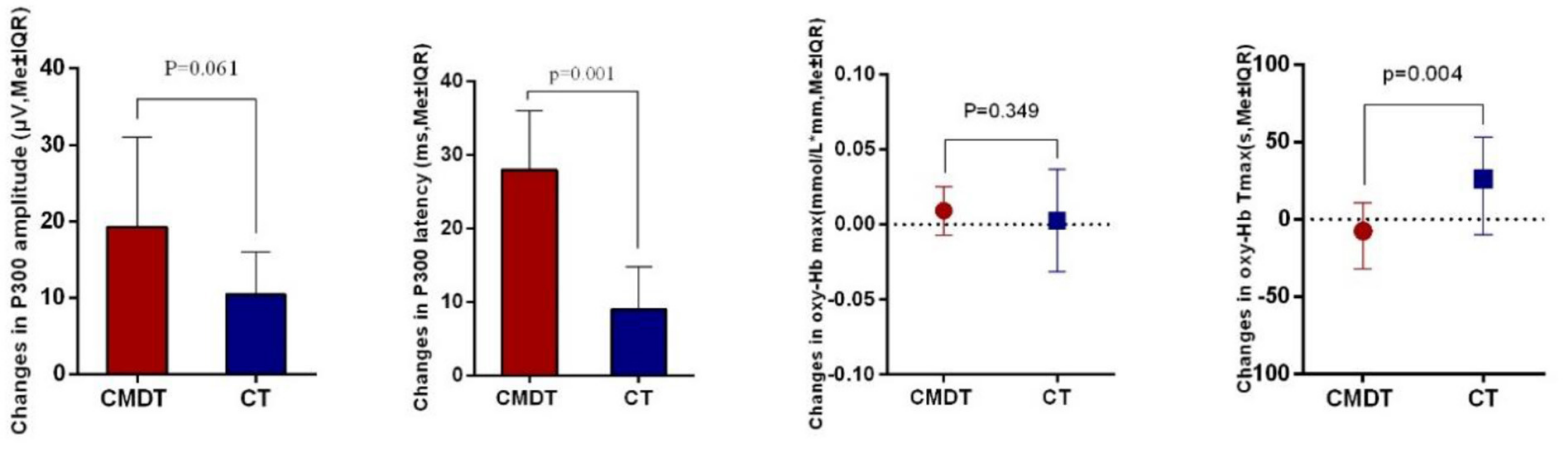
Training effect on the secondary outcome measurement.

**Figure 5:**
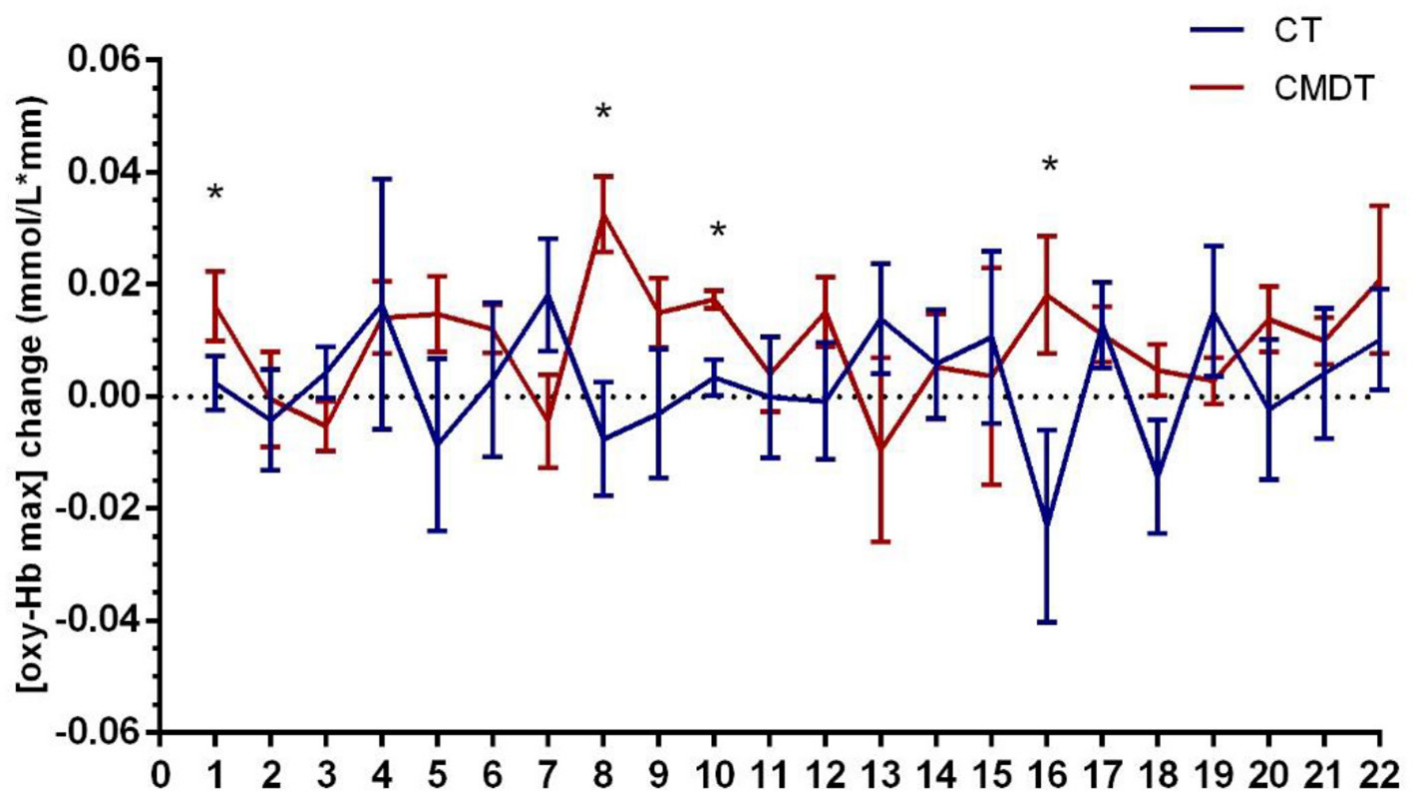
Changes in [oxy-Hb max] across 22 channels. Error bars represent one standard error of the mean. *Statistical significance.

**Figure 6:**
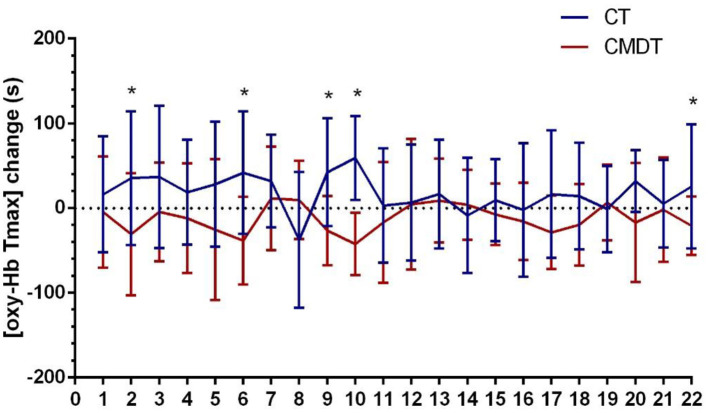
Changes in [oxy-Hb Tmax] across 22 channels. Error bars represent one standard error of the mean. *Statistical significance.

Secondary index measures included the amplitude and latency of P300 and the changing trend of oxy-Hb concentration. The results of Table 3 showed that both P300 latency and the time to the peak level of oxy-Hb (oxy-Hb Tmax) differed significantly between the two groups. The P300 amplitude in lead Fz increased by 0.2150 μV in the CT group and 0.3260 μV in the CMDT group. There was no significant difference between the two groups (*P* = 0.061). The mean P300 latency of Fz lead was shortened 11.25 ms in the CT group and 26.41 ms was shortened to the CMDT group. There was a significant difference between the two groups (*P* = 0.001). The average peak level of oxy-Hb (oxy-Hb max) in the frontal lobe increased by 0.0092 mmol/L^*^mm in the CMDT group and 0.0025 mmol/L^*^mm in the CT group. There was no significant difference between the two groups (*P* = 0.349). The average value of oxy-Hb Tmax was shortened by 11.09 s in the CMDT group and prolonged by 23.14 s in the CT group. There was a significant difference between the two groups (*P* = 0.004) (Figures 4–6).

**Table 3:** Estimated mean change and statistical significance in secondary outcome variables.

**Variables**	**Group**	**Difference (mean)**	**Z**	***P*** **-value**
P300 amplitude (μV)	CMDT	0.3260	−1.874	*P =* 0.061
	CT	0.2150		
P300 latency (ms)	CMDT	−26.41	−3.247	*P =* 0.001^*^
	CT	−11.25		
oxy-Hb max (mmol/L^*^mm)	CMDT	0.0092	−0.937	*P =* 0.349
	CT	0.0025		
oxy-Hb Tmax (s)	CMDT	−11.09	−2.846	*P =* 0.004^*^
	CT	23.14		

* Statistical significance.

### Adverse events

No study-related adverse events occurred in either the CMDT training or active control group.

## Discussion

PSCI is a functional disorder with a high incidence after stroke. The treatment of cognitive impairment after stroke has great significance for improving the quality of life of patients and reducing the social burden. The purpose of this proof-of-concept randomized trial was to investigate the efficacy of CMDT training in patients with PSCI. The strengths of our study include its active control design and use of both neuropsychological evaluation and ERP and fNIRS as outcomes.

For primary index measurement, we found that, relative to the CT, CMDT training led to a significant improvement in global cognitive function, as measured by MMSE and MoCA, by the end of the 4 week intervention. This result is consistent with findings from recent meta-analyses of CMDT training (12, 22). It shows that CMDT training has more advantages than single-cognitive training in improving cognitive impairment. Our results provide a significant update to the current understanding of the effects of CMDT training. We confirm the positive effects of combined interventions on cognition reported in previous studies (20, 23, 24).

Concerning secondary index measurement, ERP and fNIRS tests showed that, compared with CT, dual-task intervention could significantly shorten the latency of P300 and the oxy-Hb Tmax. The result of this study is an important supplement to the research on the improvement of the cognitive field of dual-task training. Previous studies have shown that MAP training (combining meditation and aerobic exercise) can improve cognitive function after stroke and increase the amplitude of P300 (25, 26). Our study also found that after CMDT training, the P300 amplitude increased, but there was no statistical difference compared with the CT group. More importantly, our results show that the P300 latency in the CMDT group is significantly shorter than that in the CT group. It is well known that the latency of P300 reflects the time it takes for the brain to respond to stimulation (27, 28). The results showed that after dual-task training, the reaction time of cognitive tasks decreased. Many studies have shown that the latency of P300 is a more sensitive index than amplitude in patients with stroke (21, 29). Some studies have found that the latency of P300 in patients with vascular cognitive impairment is significantly prolonged, but the amplitude of P300 has no significant difference from that of normal subjects (30). The results of this study confirmed that P300 latency is a sensitive index to detect the improvement of cognitive function in patients with PSCI from the point of view of cognitive improvement.

A study by Dong et al. (31) points out that oxy-Hb Tmax correlating with the reaction time means that the latency reflects the speed of cognitive processing. Our results showed that the oxy-Hb Tmax of the CMDT group was significantly shorter than that of the CT group. Therefore, the results of oxy-Hb Tmax once again confirmed that CMDT training can reduce the task-related reaction time of patients. It suggests that the brain could increase oxy-Hb more rapidly under the stimulation of the cognitive task and excite central neuronal cells, thus significantly accelerating nerve conduction velocity and cognitive processing speed (32). This is consistent with the results of ERP.

Through the analysis of the results of neurocognitive psychology, our study demonstrated that CMDT training significantly improved PSCI compared with cognitive training, and the results of fNIRS and ERP suggested that CMDT training could significantly shorten cognitive reaction time and improve cognitive processing speed. The present study was subject to several limitations. The evaluation scale of cognitive function in the outcome index of this study evaluated overall cognitive function, and the index was not detailed enough. In future experiments, evaluation methods for each cognitive domain (such as attention, memory, computing power, orientation, and executive function) could be refined to determine the specific areas in which the training method could improve cognitive function. Future research could also increase the follow-up time and explore the long-term effect of CMDT training. Owing to equipment limitations, fNIRS only collected data from the frontal lobe. Further research could collect other information from the other brain lobes.

## Conclusion

In general, compared with cognitive training, CMDT training had more obvious advantages in improving the cognitive function of PSCI patients. CMDT training shortened the reaction time of central neurons and accelerated the nerve conduction velocity to accelerate the speed of cognitive processing and improve cognitive function more effectively. Although our study demonstrated the effectiveness and safety of CMDT training in PSCI patients, more clinical trials are needed to support these findings.

## Data availability statement

The raw data supporting the conclusions of this article will be made available by the authors, without undue reservation.

## Ethics statement

The studies involving human participants were reviewed and approved by the Ethics Committee of Beijing Rehabilitation Hospital at Capital Medical University (2020ky59). The patients/participants provided their written informed consent to participate in this study.

## Author contributions

WG: study concept and design. RS: acquisition of data, analysis and interpretation, and critical revision of the manuscript for important intellectual content. XL and ZZ: analysis and interpretation. MZ and LM: acquisition of data. TL, WL, XX, and PH: critical revision of the manuscript for important intellectual content and study supervision. All authors read and approved the final manuscript.

## Funding

This study was sponsored by the National Natural Science Foundation of China (81972148) and Capital's Funds for Health Improvement and Research (2022-1-2251).

## Conflict of interest

The authors declare that the research was conducted in the absence of any commercial or financial relationships that could be construed as a potential conflict of interest.

## Publisher's note

All claims expressed in this article are solely those of the authors and do not necessarily represent those of their affiliated organizations, or those of the publisher, the editors and the reviewers. Any product that may be evaluated in this article, or claim that may be made by its manufacturer, is not guaranteed or endorsed by the publisher.
